# Shallow magma pre-charge during repeated Plinian eruptions at Sakurajima volcano

**DOI:** 10.1038/s41598-019-38494-x

**Published:** 2019-02-13

**Authors:** Naoki Araya, Michihiko Nakamura, Atsushi Yasuda, Satoshi Okumura, Tomoki Sato, Masato Iguchi, Daisuke Miki, Nobuo Geshi

**Affiliations:** 1 Department of Earth Science, Graduate School of Science, Tohoku University, 6-3 Aramaki Aza-Aoba, Aoba-ku, Sendai 980-8578 Japan; 2 Earthquake Research Institute, University of Tokyo, 1-1-1 Yayoi, Bunkyo-ku, Tokyo 113-0032 Japan; 3 Research and Development Center for Ocean Drilling Science, Japan Agency for Marine-Earth Science and Technology, 2-15 Natsushima-cho, Yokosuka, 237-0061 Japan; 4 Sakurajima Volcano Research Center, Disaster Prevention Research Institute, Kyoto University, 1722-19 Sakurajima-Yokoyama, Kagoshima, 891-1419 Japan; 5 Geological Survey of Japan, National Institute of Advanced Industrial Science and Technology, 1-1-1 Higashi, Tsukuba, 305-8567 Japan

## Abstract

Vigorous explosive eruptions that produce continuous high eruption plumes (Plinian eruptions) are generally assumed to tap a magma reservoir. The 1914 Plinian eruption at the Sakurajima volcano located on the Aira caldera rim is one such case, where the main magma reservoir was assumed to be located approximately 10 km beneath the caldera. However, we report that estimated magma storage depths immediately prior to the eruption were much shallower (0.9–3.2 km) on the basis of pressure at which volatiles within the phenocryst melt inclusions and plagioclase rims were finally equilibrated. The same is observed for two historic Plinian eruptions in 1471 and 1779. This depth is even shallower than the shallowest magma reservoir estimated from the pressure source for geodetic deformation during recent Vulcanian explosions (4 km beneath the crater). We propose that the magmas were fed from a thick conduit pre-charged from deeper reservoirs. The ground subsidence observed after 1914 within the Aira caldera may have been caused by conduit recharge following the eruption. Voluminous conduit recharge could be key to forecasting the next possible large eruption at the Sakurajima volcano.

## Introduction

Forecasting the initiation of future eruptions is a challenging but rewarding task in volcanology. A common and promising approach is to learn from pre-eruptive processes of past, similar eruptions^1–5^. Historically, the Sakurajima volcano, Kyushu, Japan, has experienced three periods of repeated Plinian eruptions of VEI 4–5 accompanied by voluminous lava effusion^6^ (AD 1471–1476, 1779–1782, and 1914–1915; Supplementary Table S1; see Supplementary Information). Eruption magnitudes and sequences are similar for all three episodes, providing us a rare opportunity to reveal the recurring pre-eruptive process from magma accumulation to discharge. An imminent concern for this volcano is if, when, and how the next eruption of similar magnitude will occur because the surface level within the Aira caldera, which subsided during the 1914–1915 eruption, has almost returned to its original pre-eruption level^7,8^, suggesting that the main magma reservoir has been almost fully recharged. Located only 7 km west of the crater of the previous Plinian eruption in 1914, Kagoshima city has a population of 600,000; thus, this is a pressing research issue.

**Figure 1:**
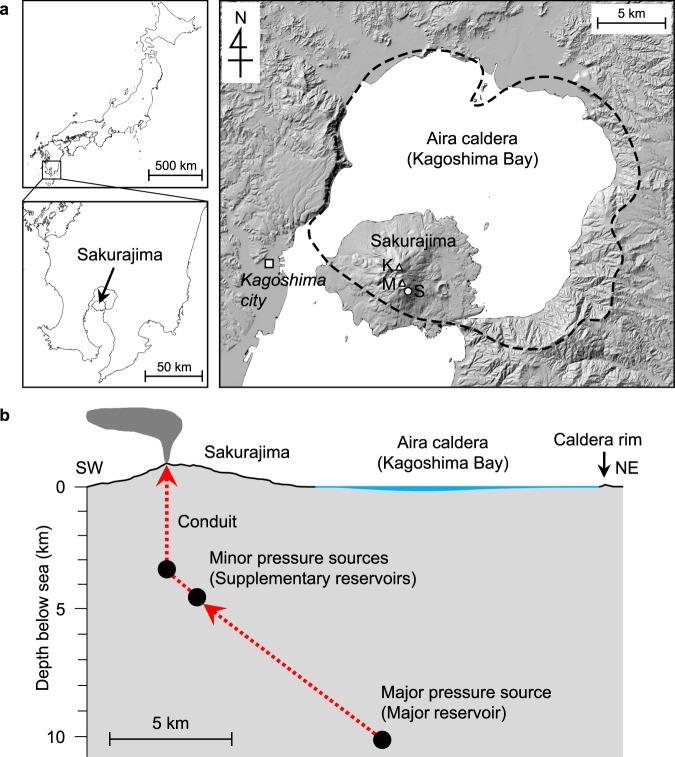
(**a**) Index map. Sakurajima is a post-caldera stratovolcano located at the rim of the Aira caldera (black dashed line). K: Kitadake summit; M: Minamidake summit; S: Showa crater. The shaded relief map was created from a 5-m resolution Digital Elevation Model provided by Geospatial Information Authority of Japan (https://fgd.gsi.go.jp/download/menu.php)^50^ and it was edited by the authors. (**b**) A schematic illustration of the present magma plumbing system of the Sakurajima volcano inferred from geophysical observations^12^. Black circles indicate pressure sources for geodetic deformation, which are assumed to correspond to the positions of magma reservoirs. The major magma reservoir beneath the Aira caldera caused significant ground subsidence after the 1914–1915 eruption^9^. Dotted red lines indicate assumed magma movement during the current Vulcanian activity.

Sakurajima volcano has been monitored intensively with seismological and geodetic networks since the early 20th century^9–13^, and a structural model of the present magma feeding system has been established. There are chiefly two pressure sources responsible for recent geodetic deformation: the main source beneath the Aira caldera at a depth of ~10 km below sea level in the Kagoshima bay and a supplementary source group at a depth of 4–6 km beneath the summit; these are interpreted to indicate locations of the major deep reservoir and minor shallow magma reservoirs, respectively^11,12^ (Fig. 1). Seismic attenuated zones accompany these reservoirs^11^. The diameter of the conduit connecting the shallowest reservoir and the summit crater is estimated to be 300–500 m at a depth of 3 km beneath the crater, based on the hypocentre distribution of volcanic earthquakes^12^.

In this study, in order to obtain volatile saturation pressure, we analysed the chemical compositions and volatile contents of >100 phenocryst-hosted melt inclusions obtained from the three historic Plinian eruptions and recent Vulcanian explosions in 1975–2010. Furthermore, we calculated H_2_O contents in matrix melts in equilibrium with plagioclase phenocryst rims based on plagioclase-hygrometers to further constrain pre-eruptive volatile contents. From these data, we demonstrate that the erupted magmas were pre-charged in the shallow conduit immediately prior to the eruptions.

## Petrological Background of Historic Eruptions at Sakurajima Volcano

Petrological studies have shown that the magmas erupted at Sakurajima since 1471 were formed via magma mixing. The bulk rock composition has shifted from dacitic to andesitic over time^14–20^ (Supplementary Table S1). Nakagawa *et al*.^16^ revealed that binary mixing of dacitic and andesitic endmembers formed the eruptive products of the 1471–1476 and 1779–1782 eruptions, while contribution from a third basaltic endmember magma is required to explain the compositions of magmas erupted since the 1914–1915 eruption. The petrochemical details of the endmember magmas are described in Supplementary Information.

## Results

### Major element compositions and microstructure of melt inclusions

**Figure 2:**
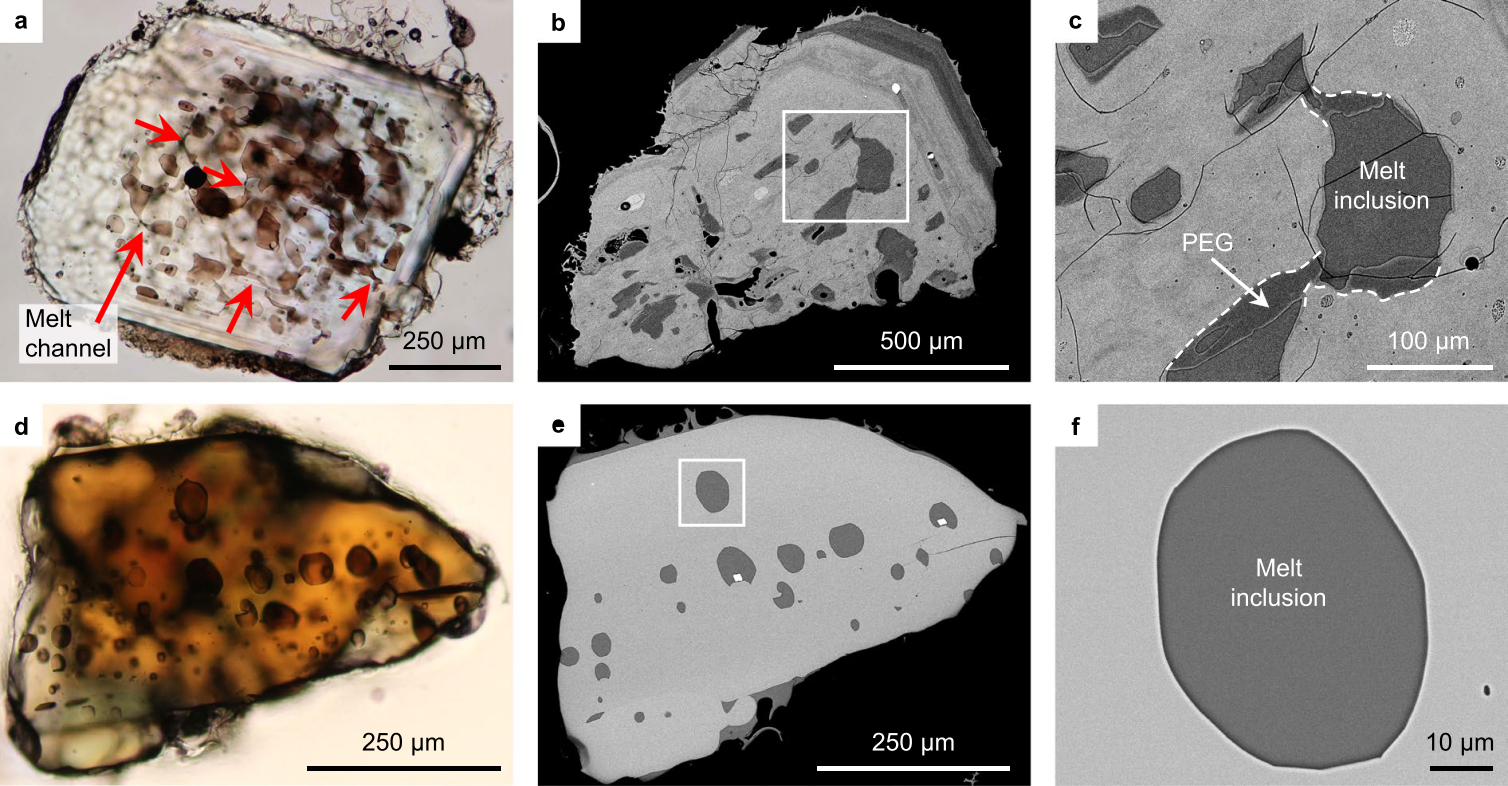
Photomicrographs (**a**,**d**: open nicol) and back scattered electron images (**b**,**c**,**e**,**f**) of representative melt inclusions in plagioclase (**a**–**c**) and pyroxene (**d**–**f**) from the 1914 Plinian pumice. (**a**,**b**) are different plagioclase crystals, while (**d**,**e**) are the same pyroxene crystal. (**c**,**f**) are enlargements of the white frames in (**b**,**e**), respectively. The plagioclase-hosted melt inclusions show a sieve texture, interconnected via narrow melt channels (red arrows in **a**). Significant post-entrapment (and pre-enclosed) growth (PEG) is observed in **c**. The dashed lines in **c** show boundaries between the original plagioclase and melt inclusion before the growth. By contrast, the pyroxene-hosted inclusions are mostly isolated (**d**–**f**). Post entrapment growth of pyroxene is scarcely observed (typically <500 nm thick).

**Figure 3:**
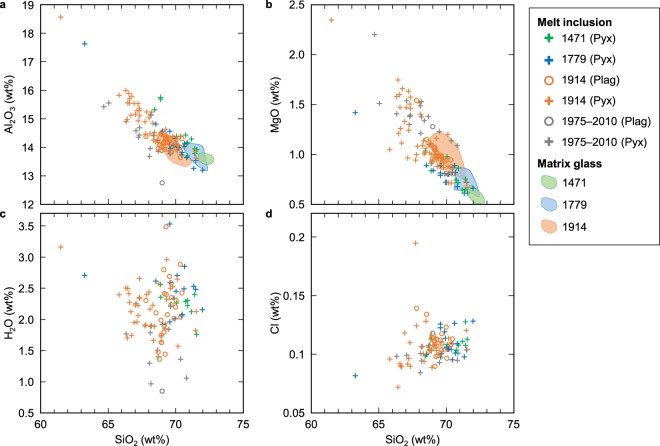
SiO_2_ variation diagrams of melt inclusion compositions. The major element composition (**a**: Al_2_O_3_, **b**: MgO) of the most silica-rich inclusions coincides with that of matrix groundmass matrix glass for each Plinian eruption. (**c**) H_2_O contents do not exhibit a systematic correlation with SiO_2_ contents. (**d**) Cl contents of melt inclusions in plagioclase (Plag) do not correlate with SiO_2_ contents, whereas those in pyroxene (Pyx) have a weak negative correlation.

We analysed 148 melt inclusions hosted by plagioclase, orthopyroxene, and clinopyroxene, the major dominant phenocrystic phases (Fig. 2). The major element compositions of melt inclusions are shown in Fig. 3a,b and Supplementary Table S2.

The inclusion-hosting phenocrysts, both plagioclase and pyroxene, have a wide compositional range (Supplementary Fig. S1) as they were mostly derived from the endmember magmas of mixing, although some were formed after mixing. It is notable that plagioclase-hosted melt inclusions have a narrower compositional range with a higher minimum SiO_2_ content (68–70 wt%) than the pyroxene-hosted melt inclusions (61–72 wt%) (Fig. 3). This is consistent with the contrasting microstructure of melt inclusions, i.e., most plagioclase-hosted melt inclusions show a sieve texture^21^, whereas pyroxene-hosted melt inclusions are generally isolated with a rounded shape (Fig. 2). Such melt inclusions in plagioclase are formed by partial dissolution upon magma mixing^21^. They are actually melt channels that were connected to the phenocryst surface until a later stage of crystal growth induced by decompression to the final emplacement depth, which then shut off these channels. Conversely, pyroxene-hosted melt inclusions were trapped and enclosed at various stages in the endmember magmas and upon final magma mixing. In backscattered electron images, post-entrapment (and pre-enclosed) growth of host plagioclase is significant, whereas post-entrapment (and post-enclosed) growth of host pyroxenes is generally less than 500 nm thick (Fig. 2). This is consistent with the water pressure dependence of the liquidus temperature being smaller for pyroxene than for plagioclase, along with the fact that the inclusion compositions are not controlled by their host minerals; namely, they are scarcely affected by post-entrapment growth of host pyroxene and plagioclase (Fig. 3a,b).

### Volatile contents of melt inclusions

**Figure 4:**
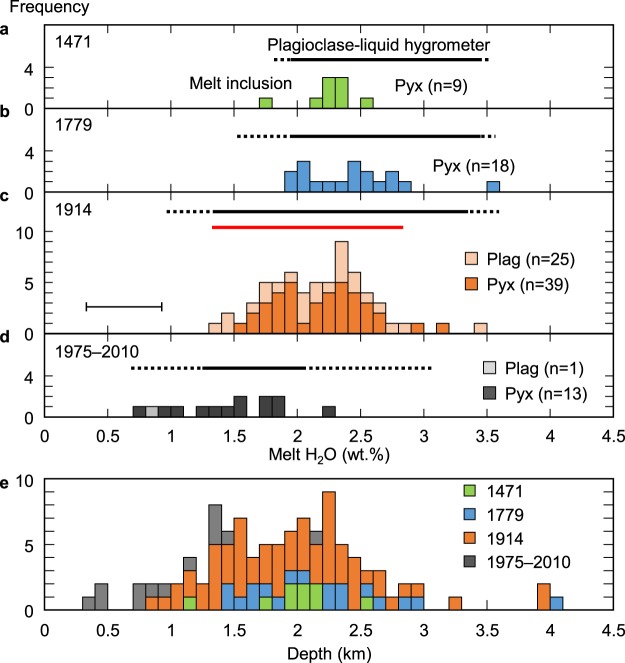
Frequency distribution of melt inclusion H_2_O contents and estimated depths. H_2_O contents in melt inclusions from the Plinian eruptions in 1471 (**a**), 1779 (**b**), and 1914 (**c**), and Vulcanian explosions in 1975–2010 (**d**) measured by FT-IR reflectance spectroscopy. Black and red bars represent the melt H_2_O contents calculated with a plagioclase-melt hygrometer^33^. See Supplementary Fig. S3 for details. The analytical accuracy of the FTIR analysis is shown in **c**. (**e**) Magma storage depths estimated from the final equilibrated pressure of H_2_O contents in melt inclusions calculated with the H_2_O solubility model^32^.

H_2_O contents in the melt inclusions from the Plinian pumices are 1.4–3.5 wt%, >95% of which are within 1.4–3.0 wt% (Figs 3c and 4, Supplementary Table S2). We additionally analysed the volatile contents of 35 pyroxene-hosted melt inclusions from the 1914 eruption with transmission FT-IR, which has a better accuracy for H_2_O measurement and a lower detection limit for CO_2_ (~10 ppm). The H_2_O contents measured by transmission and reflectance techniques show good agreement with each other (Supplementary Fig. S2). CO_2_ contents of 22 of 35 inclusions are below the detection limit. The highest CO_2_ content is 29 ppm. Its effect on pressure estimation from H_2_O solubility is less than ~5 MPa. Compared to the widely-ranging major element compositions of melt inclusions in pyroxene, their H_2_O contents are concentrated in a relatively narrow range and exhibit no systematic correlation with major element composition, similar to those in plagioclase (Fig. 3c, Supplementary Fig. S1).

Melt inclusions in the recent Vulcanian explosion products have clearly lower H_2_O contents (0.7–2.3 wt%) than those in the Plinian pumices (Fig. 4). We interpret that the melt inclusions in pyroxene and plagioclase phenocrysts re-equilibrated (i.e., degassed) during magma ascent and emplacement in a shallow conduit. Hydrogen diffusion in crystals^22–28^ and molecular H_2_O transport along some fast diffusion paths such as microcracks, dislocations, and cleavages^29,30^ in the host plagioclase and pyroxene are possible mechanisms of degassing. Considering this, we assume that the H_2_O content of the melt inclusions from Plinian pumices had enough time to re-equilibrate at their final storage depth upon entrapment^31^.

Among the crystals of the Plinian pumices, multiple melt inclusions were analysed in a single crystal for eight plagioclases and eight pyroxenes. Their H_2_O contents were generally homogeneous, except for four plagioclase crystals. In these four plagioclase crystals, the range of H_2_O contents of the melt inclusions exceeded the range of analytical accuracy, despite the assumption that all the melt inclusions in a crystal should have the same H_2_O content after re-equilibration. In such a case, the lowest H_2_O contents reduced to 1.4 wt%. The variation in H_2_O contents in a single phenocryst may be explained by syn-eruptive leakage. Based on this observation, we conclude that the majority of melt inclusions preserve the H_2_O content at the time of re-equilibration prior to eruption, but some could have leaked syn-eruptively, reducing the H_2_O content to 1.4 wt%. This view is consistent with the fact that magma ascent rates in Plinian eruptions are generally much higher than those in Vulcanian explosions^18^.

In order to verify the H_2_O re-equilibration model, we analysed Cl contents in the melt inclusions, which are roughly proportional to the H_2_O content in the magma although less likely to decrease due to lower solubility and smaller diffusivity in the host crystals than hydrogen and water^25^. Cl contents of melt inclusions in plagioclase from the Plinian pumices are 0.09–0.14 wt%, with no correlation to SiO_2_ content, while those in pyroxene are 0.07–0.19 wt%, having a weak positive correlation with SiO_2_ content (R = 0.28) (Fig. 3d). This is consistent with the major element trends resulting from the contrasting origin of melt inclusions; i.e., those in pyroxene were entrapped at various stages in the endmember and mixed magmas, whereas a significant proportion of melt inclusions in plagioclase were connected to the surrounding melt until the final stage. As Cl content re-equilibration via diffusion through host crystals is supposed to be much slower than H_2_O^25^, the original Cl content at the time of entrapment in pyroxene should be preserved. In fact, in contrast to H_2_O, the Cl contents of melt inclusions from Vulcanian explosion products have a mostly similar range to those from Plinian pumices (Fig. 3d).

The three historic Plinian eruptions show a similar range of H_2_O contents in the melt inclusions. This strongly suggests that magmas of these eruptions started ascending from a common storage region. We note that the melt composition dependence of H_2_O solubility is negligible at this low pressure (less than ca. 0.3 wt% for 61–72 wt% SiO_2_ based on the solubility model of Zhang *et al*.^32^) and melt inclusion compositions are similar among these eruptions.

### Estimation of melt H_2_O content from plagioclase-melt equilibria

**Figure 5:**
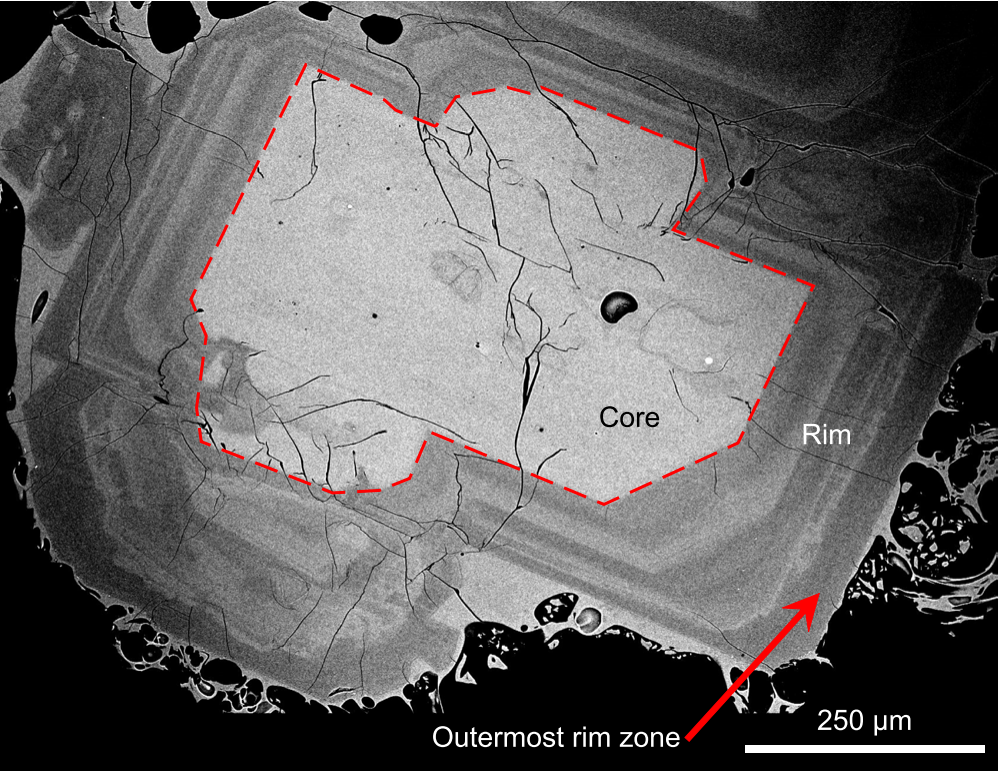
Back scattered electron images of a representative plagioclase phenocryst from the 1914 Plinian pumice. The plagioclase has a calcic core and a thick (~200 μm) relatively sodic rim with oscillatory zoning. The outermost homogeneous zone with a thickness of >10 μm (typically 20–30 μm) was analysed for the hygrometer calculation.

The H_2_O contents of melt (i.e. glassy matrix of groundmass) that equilibrated with the outermost rim zone of plagioclase phenocrysts (Fig. 5) were estimated using plagioclase-melt hygrometers (Fig. 4, Supplementary Fig. S3). The outermost rim zone, which is assumed to have grown near the final magma emplacement depth, is melt inclusion-free, and has a typical thickness of 20–30 μm and a broad but unimodal compositional distribution with a peak at An_53–60_ (Supplementary Fig. S4). The melt channels that were connected to the plagioclase surface should have become completely enclosed at this stage. Judging from the absence of groundmass microlite and well-faceted phenocryst surfaces without mineral inclusions, the phenocrysts are assumed to have formed their outermost rim zone near the final storage depth and exhibited minimal growth during rapid magma ascent upon Plinian eruption. Therefore, major element compositions of phenocryst rims and adjacent groundmass glass are expected to reflect the final magma storage pressure immediately prior to the onset of Plinian eruptions. For the Vulcanian samples, microlite poor groundmass interstices and plagioclase phenocryst rims lacking microlite-stage growth are rarely found and were measured to estimate the final magma emplacement depths. The compositional pairs of the outermost rim zone and adjacent groundmass glass are given in Supplementary Table S3.

To calculate H_2_O contents, we used the latest plagioclase-melt hygrometer^33^. Details of the calculation procedure are given in Methods. The estimated pre-eruptive melt H_2_O contents are 1.8–3.5, 1.5–3.6, and 1.0–3.6 wt% for the 1471, 1779 and 1914 Plinian eruptions, respectively, and 0.7–3.1 wt% for the Vulcanian explosions (Fig. 4). H_2_O contents calculated for the Plinian eruptions agree well with the H_2_O contents of melt inclusions directly measured with FT-IR. This supports our conclusion that most of the H_2_O contents of melt inclusions indicate the final equilibration pressure corresponding to the magma storage depth just prior to the Plinian eruptions and were scarcely affected by syn-eruptive degassing after the onset of rapid magma ascent leading to fragmentation. On the other hand, FT-IR data of the Vulcanian samples are slightly but systematically lower than the hygrometer estimates. This indicates that growth of plagioclase rims did not catch up with the magma ascent and decompression in a shallow conduit that led to diffusive degassing of the melt inclusions immediately prior to the Vulcanian explosions.

## Discussion

Silicic arc magmas are generally H_2_O-saturated in the middle to upper crust^34^. In the uppermost crust conditions studied in this study, magmas are expected to have been H_2_O-saturated. In fact, the previous experimental study on the phase equilibrium of the 1914 Plinian eruption determined the pre-eruptive melt H_2_O content to be 3.3 wt%^35^. This H_2_O content matches well with the results of this study. Although there has been no phase equilibrium experiment on the 1471 and 1779 magmas, these eruptions are likely to have similar pre-eruptive melt H_2_O contents, because the chemical compositions of matrix glasses and plagioclase rims in the 1471 and 1779 pumices are not significantly different from those of the 1914 pumices (Fig. 3a,b and Supplementary Fig. 4). Therefore, it is reasonable to assume that the pre-eruptive magmas of the historic Plinian eruptions were H_2_O-saturated.

More than 95% of melt inclusions from the historic Plinian eruptions show volatile saturation pressure ranges from 20–72 MPa, corresponding to depths of 0.9–3.2 km below the surface (Fig. 4; Methods). A few melt inclusions show relatively high pressure up to 92 MPa, which corresponds to depths of 4.1 km. An error in the depth estimation due to FT-IR analytical accuracy is estimated to be <0.7 km. Therefore, the final magma storage depths estimated from most of the melt inclusions from historic Plinian eruptions are shallower than the shallowest geophysical estimate of present magma reservoirs (4 km)^12^.

An important finding of this study is that the repeated Plinian eruption magmas were fed from very shallow depths (mainly 0.9–3.2 km beneath the crater; Fig. 4) corresponding to the conduit from the present shallowest magma reservoir. Based on the hypocentre distribution of volcanic earthquakes from the 1970’s–80’s, the estimated conduit diameter at depths of 0.9–3.2 km is 0.3–0.5 km^12^. Assuming a cylindrical conduit shape, its volume in this depth range is calculated to be 0.2–0.5 km^3^. The estimated tephra volumes of the historic Plinian eruptions are 0.8, 0.3, and 0.5 km^3^ for the 1471, 1779, and 1914 eruptions, respectively^6^ (Supplementary Table S1). Assuming a pumice vesicularity of 74 vol%, which is an average vesicularity of the 1914 pumices^36^, the dense-rock equivalent magma volumes are 0.1–0.2 km^3^. This shows that the volume of the present conduit can be large enough to supply most of the magma erupted during the Plinian phases. This calculation also indicates that at least some of the magmas that effused as lava flows following the Plinian eruptions should have been fed from the shallowest magma reservoir beneath the conduit, although their pre-eruptive storage depths have not been estimated because their melt inclusions and groundmass underwent significant degassing and crystallisation. Formation of this thick conduit might be related to the crustal structure beneath the Sakurajima volcano, namely, its location at the rim of the Aira caldera. It is known that many Cretaceous–Paleogene, shallow and small scale (a few hundred metres thick) intrusive rocks (porphyry and porphyrite) are exposed around caldera rims in the Chugoku district, SE Japan^37^.

**Figure 6:**
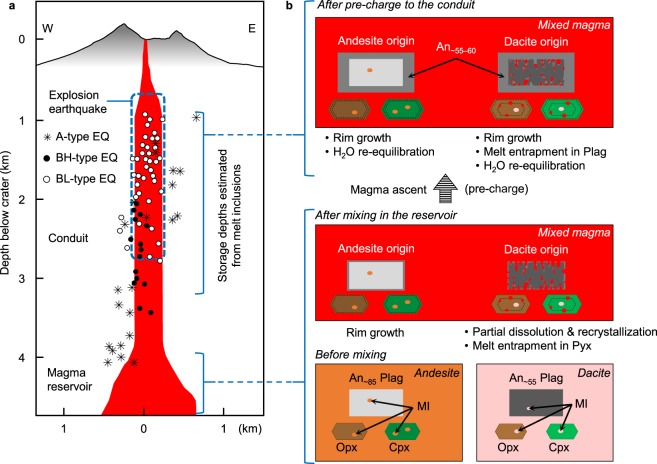
Magma pre-charge processes recorded in phenocryst microstructures in the historic Plinian eruptions. (**a**) Hypocentre distribution of volcanic earthquakes of recent Vulcanian explosions^12^ that coincides with the final mama storage depths of the historic Plinian eruptions. A-type earthquakes (EQ), and BL- and BH-types are caused by brittle fracture of rocks and fluid movement, respectively^11^. (**b**) Pyroxene-hosted melt inclusions, trapped at various timings, were re-equilibrated after pre-charge to the conduit. Melt channels in plagioclase formed by partial dissolution upon magma mixing were enclosed in the conduit as melt inclusions. See Supplementary Information for details of mixing endmembers.

This study indicates that magmas were loaded to the shallow conduit prior to each Plinian eruption since the 15^th^ century (Fig. 6). This magma pre-charge should have occurred well before the eruption to allow for growth of the 20–30 μm outermost rims of plagioclase phenocrysts in the feeder conduit. Considering the growth rate of plagioclase in previous decompression-crystallisation experiments (~10^−11^ m/s in a rhyolitic melt at 900 °C and 50 MPa after rapid decompression from 125 MPa^38,39^), we estimate that the timescale of rim growth is more than a few tens of days. Judging from our observation that melt inclusions in the Vulcanian products are more degassed than in the Plinian pumices, the degassing of melt inclusions may proceed in the shallow conduit within the intervals of Vulcanian explosions, i.e., as short as several hours. Therefore, re-equilibration of the H_2_O content of melt inclusions at the final storage depth occurs rapidly enough to enable growth of the outermost rim of plagioclase. Using the model of Qin *et al*.^22^ and a diffusivity value of 10^−11^–10^−12^ m^2^/s^40^, we calculated the duration required for 90% re-equilibration of H_2_O content in typical sized spherical melt inclusions as less than 5 days, demonstrating the plausibility of re-equilibration (Supplementary Fig. S5).

Our petrological analyses demonstrate that the magma erupted as pumice from the Plinian eruptions in 1914, 1779, and 1471 was stored in the shallow conduit, not in the main reservoir ~10 km beneath the Aira caldera. We propose that the ground subsidence observed around the Aira caldera after the 1914–1915 activity^9^ was caused by magma recharge from the main reservoir to the shallower feeding system following the eruption. The present surface level has been approaching the level achieved before the 1914–1915 eruption^7,8^, suggesting that the main reservoir beneath the Aira caldera has been almost fully recharged and thus has the potential for another eruption. Given the fact that Plinian and lava flow eruptions have been repeated three times in a similar magnitude and sequence, it is reasonable to assume that the next eruption would be preceded by loading of ~0.2–0.5 km^3^ magma into the present shallow conduit. Our study demonstrates that intensive monitoring of the shallow conduit in addition to deep magma reservoirs is crucial for detecting the signs of future large-scale eruptions.

## Methods

### Sampling points and sample preparation

Fallout pumices of the 1471 eruption were collected from an outcrop at Yunohira, 3 km northwest of the Minamidake summit. The samples were collected from the lower portion of the fall deposit (approximately 30 cm from the bottom of the deposit of >1.1 m total thickness). The fallout pumices of the 1779 and 1914 eruptions were sampled from an outcrop at Nagasakibana, 5 km east of the Minamidake summit. The 1779 eruption pumice was sampled from the lower part (approximately 15 cm from the bottom) of a non-welded deposit with a total thickness of 1.0 m. The 1914 eruption pumice was collected from several heights of a deposit 1.8 m thick. Ejecta of recent Vulcanian explosions were sampled from various sites near the Sakurajima volcano immediately after each Vulcanian explosion (Supplementary Table S4); these ejecta consist of pumice clasts and juvenile fragments. For further analyses, non-oxidised clasts a few cm in diameter were selected. These should have been quenched upon magma fragmentation; thus, degassing of melt inclusions after fragmentation^26^ should be negligible even if cracking of host crystals had occurred.

We gently crushed the pumices from the Plinian eruptions and separated the phenocrysts, which were mounted in epoxy resin and polished until melt inclusions were exposed. Vulcanian ejecta were mounted in resin without crushing because of the limited number of the samples. In addition to the mounted samples, thin sections were made for both Plinian and Vulcanian samples for observation by optical microscope and SEM.

Lavas from the 1914–1915 eruption were collected at several locations in the eastern part of the Sakurajima volcano. Thin sections of the lava samples were made for observation by optical microscope and SEM.

### Electron microprobe analysis

Chemical compositions of melt inclusions and their host pyroxenes adjacent to the melt inclusions were analysed by wavelength dispersive X-ray spectroscopy (WDS) with JEOL JXA-8800M at Tohoku University. In the analysis, the accelerating voltage was set to 15 kV, the probe current was 10–15 nA (melt inclusions) and 15 nA (pyroxenes), and peak counting times were 10–20 s (melt inclusions) and 20 s (pyroxenes). Melt inclusions were analysed with a defocused beam (5–20 μm) and pyroxenes were analysed with a focused beam. To minimise Na loss from melt inclusions during the analysis, Na was analysed in the first sequence.

Plagioclases and matrix glass in pumices were analysed by an energy dispersive X-ray spectroscopy (EDS) equipped with a scanning electron microscope (SEM) (Hitachi-S3400N with Oxford-INCA system and JEOL JSM-7100F with JED-2300 system) at Tohoku University. The accelerating voltage and beam current were set to 15 kV and 1 nA, respectively. The total live counting time was 100 s for the Oxford-INCA system and 50 s for JED-2300, depending on the detector size. Plagioclase was analysed with a focused beam or area analyses (~5 × 5 μm^2^) and matrix glasses were analysed with area analyses (10 × 10 to 30 × 30 μm^2^). We used EDS for analyses of plagioclase and matrix glass because it has the advantages of higher space resolution, flexible setting of the analytical area, and minor Na-loss due to the lower beam current than WDS.

### FT-IR reflectance spectroscopy

The H_2_O contents of melt inclusions were measured by Fourier transform infrared (FT-IR) reflectance microspectroscopy at the Earthquake Research Institute, University of Tokyo. We used an evacuation FT-IR system composed of a JASCO FT/IR-660 Plus spectrometer and JASCO IRT-30 microscope. IR spectra were obtained with 20 × 20 to 85 × 85 μm^2^ rectangular apertures by accumulating 200 to 1000 scans. The wavenumber resolution was 4 cm^−1^. For determining H_2_O content, we followed the procedure of Yasuda^41^ and used the calibration curve for a rhyolitic melt. The analytical accuracy of the FT-IR reflectance spectroscopy was <0.3 wt%.

### FT-IR transmission spectroscopy

H_2_O and CO_2_ contents of pyroxene-hosted melt inclusions in the 1914 pumice were determined for doubly polished thin sections with an FT-IR transmission microspectrometer (Thermo Scientific Nicolet iN10) at Tohoku University. The IR spectra were obtained with 20 × 20 to 40 × 40 μm^2^ rectangular apertures by accumulating 100 to 1000 scans. The wavenumber resolution was 4 cm^−1^. To determine H_2_O and CO_2_ contents, the peak height of the absorption band at 3550 cm^−1^ and the peak area of the absorption band at 2350 cm^−1^ were measured, respectively. H_2_O and CO_2_ contents were calculated using the Lambert-Beer law using the measured absorption peak height or area, thickness of doubly polished thin sections, glass density, and the molar absorption coefficient. The thickness was measured with a Mitutoyo digital micrometer. The glass density was calculated from the relationship between glass density and H_2_O content^42^. A molar absorption coefficient of 68 ± 1 L mol^−1^ cm^−1^ (ref.^43^) and 16000 L mol^−1^ cm^−2^ (ref.^44^) were used for H_2_O and CO_2_, respectively.

### Estimation of temperature, H_2_O saturation pressure, and depth

We calculated magma temperature and volatile saturation pressure iteratively using mineral-melt thermometers and a volatile solubility model. These were applied to melt inclusion and host phenocryst pairs, and optimal magma temperature and volatile saturation pressure were obtained (Supplementary Table S2). For the temperature calculation, we used the orthopyroxene- and clinopyroxene-melt thermometers^45^ and the plagioclase-melt thermometer^33^. The saturation pressure was calculated using the pure H_2_O solubility model^32^ because the CO_2_ content in melt inclusions is very low, as described previously. The melt H_2_O contents measured by FT-IR reflectance spectroscopy were used for the calculation. The obtained magma temperatures are as follows: 907–979 °C for the 1471 eruption, 914–993 °C for the 1779 eruption, 922–1031 °C for the 1914 eruption, and 962–1028 °C for the recent Vulcanian explosions (Supplementary Table S2). The temperature range obtained for the 1914 eruption was consistent with a previous estimate by a two-pyroxene thermometer (940–1010 °C)^46^.

As reported for other arc volcanoes^47^, H_2_O contents in plagioclase-hosted melt inclusions have a negative correlation with temperatures estimated using a plagioclase-melt thermometer (Supplementary Table S2). This negative correlation may be produced by an increase in temperature due to latent heat of crystallisation and/or by H_2_O leakage (degassing) of melt inclusions without re-equilibration of plagioclase^47^. As crystallisation of plagioclase + orthopyroxene + magnetite results in a temperature increase of 3.2 °C per 1% crystallisation^48^, up to 30% crystallisation is required to explain the temperature range by the release of latent heat. The investigated Plinian pumices are, however, microlite-free and the modal compositions of phenocrysts do not vary significantly (9–17 vol%)^36^. Therefore, the negative correlation cannot be explained solely by the effect of latent heat, and degassing of melt inclusions without the growth of plagioclase is required, as discussed in the main text.

We estimated magma storage depths by converting the volatile saturation pressures of melt inclusions to depths assuming lithostatic pressure and crustal density of 2300 kg/m^3^, which is the average density around the Sakurajima volcano obtained by gravity surveys^49^.

### Melt H_2_O calculation with plagioclase-melt hygrometers

We used the latest plagioclase-melt hygrometer^33^. We also tested another hygrometer by Putirka^45^ for comparison (Supplementary Fig. S3). In the hygrometers, magma temperature and pressure are assumed, and temperature affects the estimation of melt H_2_O content significantly^47^. Temperature was estimated from mineral-melt equilibria between melt inclusion and host crystal pairs (Supplementary Table S2). When a plagioclase phenocryst contains a melt inclusion from which the temperature is estimated, the estimated temperature was applied to its rim and groundmass glass pairs. When a plagioclase phenocryst contains multiple melt inclusions that yielded temperatures, their average value was used. For phenocryst rim-groundmass glass pairs lacking melt inclusions for temperature estimation, we applied the maximum and minimum estimated temperature for each eruption to cover the possible temperature range of the pre-eruptive magmas: 907 and 979 °C for the 1471 eruption, 914 and 993 °C for the 1779 eruption, 922 and 1031 °C for the 1914 eruption, and 962 and 1028 °C for the recent Vulcanian explosions. Assuming that magma was saturated for pure H_2_O fluids, we iteratively calculated the melt H_2_O contents and saturation pressure with the hygrometer models and H_2_O solubility model^32^ and obtained the optimal values.

## Supplementary information

Supplementary Information

Supplementary Table 2 and 3

## Data Availability

The authors declare that all the data supporting the findings of this study are available within the paper and its Supplementary Information files.
