# Supplementary material for: Shallow magma pre-charge during repeated Plinian eruptions at Sakurajima volcano
Source: Sci Rep. 2019 Feb 13;9:1979. doi: 10.1038/s41598-019-38494-x (PMC6374428; doi:10.1038/s41598-019-38494-x)
Supplement: Supplementary file 1 — Supplementary Information [file 41598_2019_38494_MOESM1_ESM.docx]

Shallow magma pre-charge during repeated Plinian eruptions at Sakurajima volcano

Naoki Araya^1*^, Michihiko Nakamura^1^, Atsushi Yasuda^2^, Satoshi Okumura^1^, Tomoki Sato^1,3^, Masato Iguchi^4^, Daisuke Miki^4^, Nobuo Geshi^5^

^1^Department of Earth Science, Graduate School of Science, Tohoku University, 6-3 Aramaki Aza-Aoba, Aoba-ku, Sendai 980-8578, Japan

^2^Earthquake Research Institute, University of Tokyo, 1-1-1 Yayoi, Bunkyo-ku, Tokyo 113-0032, Japan

^3^Research and Development Center for Ocean Drilling Science, Japan Agency for Marine-Earth Science and Technology, 2-15 Natsushima-cho, Yokosuka 237-0061, Japan

^4^Sakurajima Volcano Research Center, Disaster Prevention Research Institute, Kyoto University, 1722-19 Sakurajima-Yokoyama, Kagoshima 891-1419, Japan

^5^Geological Survey of Japan, National Institute of Advanced Industrial Science and Technology, 1-1-1 Higashi, Tsukuba 305-8567, Japan

Correspondence to: Naoki Araya, n.araya@dc.tohoku.ac.jp

**Supplementary Figures**

**Supplementary Figure S1**. Relationship between melt inclusion compositions (**a**, **c**: SiO_2_, and **b**, **d**: H_2_O contents) and solid solution compositions of their host phenocrysts (**a**, **b**: Mg# of host ortho- and clino-pyroxenes, and **c**, **d**: anorthite content of plagioclase).

**Supplementary Figure S2**. Comparison between analytical results of reflectance and transmission FT-IR spectroscopy. Dotted line is the 1:1 line indicating perfect agreement.

**Supplementary Figure S3**. Frequency distribution of melt H_2_O contents calculated by applying the plagioclase-melt hygrometers of Waters and Lange (2015) and Putirka (2008) to the outermost rim zone of plagioclase phenocrysts and matrix glass pairs. When the plagioclase phenocryst contains melt inclusions from which the temperature is estimated, the estimated temperature (T_MI_) was used for the calculation (red histograms and the red solid line showing their range). Otherwise, minimum and maximum temperatures for each eruption, estimated from melt inclusions and their host phenocryst compositions, were used to cover the possible range of pre-eruptive magmas (dark grey: minimum temperature, light grey: maximum temperature). The black solid and dotted lines represent melt H_2_O ranges from the lowest to highest and from peak to peak, respectively. These red and black lines are shown in Figure 4. The standard error estimate (SEE) of the hygrometer models is shown in the left upper part of the panel of the 1471 eruption.

**Supplementary Figure S4**. Frequency distribution of anorthite content (An) of the outermost rim zone of plagioclase phenocrysts. All eruptions show a broad but unimodal distribution with a peak An increasing from 53 to 60 with time.

**Supplementary Figure S5**. Re-equilibration timescales of H_2_O contents in spherical melt inclusions with a radius of 25 μm calculated using the model of Qin *et al.* (1992). r_host_ is the radius of a spherical host phenocryst. Calculations were made for two diffusivity values: *D*=10^-12^ m^2^/s (lower horizontal axis) and *D*=10^-11^ m^2^/s (upper horizontal axis), which correspond to hydrogen diffusion in pyroxene at 1000 °C (Farver, 2010) and possible molecular H_2_O diffusion through microcracks in plagioclase (Hamada & Fujii, 2007). *k* is the apparent partitioning coefficient of H_2_O between melt and crystals including the effect of fast diffusion paths.

**Supplementary Tables**

Supplementary Tables S2 and S3 are located within a separate Excel file.

**Eruption history of Sakurajima volcano**

Sakurajima volcano is located at the southern rim of the Aira caldera, Kyushu, southwest Japan. Two peaks aligned north-south exist at the summit (Kitadake and Minamidake). Kobayashi *et al.* (2013) reported that three historic eruptions (1471–1476, 1779–1782, and 1914–1915 eruptions) occurred with similar eruption sequences, i.e., Plinian eruptions followed by lava effusion (Supplementary Table S1). The estimated eruptive volumes of these eruptions are 0.8–2.0 km^3^ as a dense rock equivalent (DRE) volume, which include the apparent volumes of the Plinian tephras (0.3–0.8 km^3^) (Kobayashi *et al.*, 2013). In 1946, a lava flow eruption with a DRE volume of 0.2 km^3^ occurred from the Showa crater located on the eastern slope of Minamidake. Since 1955, Vulcanian explosions have occurred at the Minamidake summit crater. Eruptions also began at the Showa crater in 2006.

Precursory phenomena and the sequence of the main eruption phase in 1914–1915 were recorded in detail (e.g. Omori, 1914; Koto, 1916) and recently revisited by Yasui *et al.* (2006). Major precursory phenomena leading to the Plinian eruption include emission of volcanic gases at Arimura, 3 km south from the Minamidake summit on September 17, 1913, which killed two people; a decrease in the water level of wells from November to December, 1913; frequent earthquakes and slope collapses since January 9, 1914; overflow of hot ground water in coastal areas, and steam emission from the summit and flank of Minamidake from 08:00 to 09:00 on January 12, 1914. The Plinian eruption started from the western and eastern flanks at around 10:00 on January 12, 1914, and lasted for 36 h (Yasui *et al.*, 2006). The eruption style then shifted to lava flows, which continued for one and a half years. The 1914–1915 eruption was accompanied by ground subsidence of up to ~1 m around the circumference of the Aira caldera (Omori, 1916).

**Petrography of the studied samples**

The phenocryst assemblage common to eruptive materials after the 1471 eruption consists of plagioclase, orthopyroxene, clinopyroxene, and magnetite. Olivine phenocryst is occasionally found in eruptive materials after the 1914 eruption. Ilmenite occurs as a phenocryst only in pumices of the 1471 eruption. Pumices of the Plinian eruptions are almost microlite-free, except for those erupted from the western flank craters in 1914. The erupted materials of the Vulcanian explosions are less vesiculated and their groundmass has much higher microlite and nanolite (Mujin and Nakamura, 2014; Mujin *et al.*, 2017) crystallinity than the Plinian pumices. Juvenile lithic fragments of the Vulcanian explosions commonly contain a significant amount of nanolites, while ultrananolites (Mujin *et al.*, 2017) have not been observed in the groundmass interstices.

34% of the pyroxene-hosted melt inclusions and 30% of the plagioclase-hosted melt inclusions of the Plinian pumices include small bubbles (<10 μm in diameter), which appear to be shrinkage bubbles formed for no volatile-leaked melt inclusions. Melt inclusions lacking shrinkage bubbles have a similar frequency distribution of volatile contents to that of shrinkage bubble-bearing melt inclusions. 9% of plagioclase-hosted melt inclusions in Plinian pumice include a large bubble up to 100 μm in diameter. These large bubbles are not thought to have been formed by expelling coexisting melt along cracks in the host crystals during decompression. Instead, they were likely included at the time of melt inclusion entrapment because the presence of large bubbles has no correlation to volatile contents in the melt inclusions.

**Endmember magmas of historic eruptions**

Yanagi *et al.* (1991) reported bulk rock and phenocryst compositions of lavas from the historic eruptions of 1471–1476 to 1946. The bulk rock composition became gradually mafic (from 67 to 60 wt% SiO_2_ content). The anorthite contents (An=Ca/(Na+Ca) in mol%) of plagioclase cores show bimodal distributions with peaks at approximately 58 and 85, which are almost constant throughout this period. The relative proportion of high An plagioclase has increased with time. Because magmatic enclaves included in the 1779–1782 and 1914–1915 lavas contain abundant An_80–90_ plagioclase phenocrysts, the enclaves are regarded as quenched products of the mafic endmember magma, with an estimated SiO_2_ content of ~52 wt%. They also reported that the enclaves contain orthopyroxenes with core Mg numbers (Mg#=Mg/(Fe+Mg) in mol%) of 75.

Nakagawa *et al.* (2011) conducted more detailed bulk rock and phenocryst composition analyses for the historic eruptions, including pumices and lavas, and Vulcanian explosions since 1955. They found that the eruptive materials of the 1914–1915 and Vulcanian explosions have a different bulk rock trend from that of 1471–1476 and 1779–1782 eruptions. Core anorthite contents of plagioclase phenocrysts have a bimodal distribution with peaks at approximately 55 and 85, as reported by Yanagi *et al.* (1991). Both orthopyroxene and clinopyroxene phenocrysts show unimodal distributions with a peak at Mg# = ~65 and ~70, respectively. Olivine phenocrysts with a compositional peak at a forsterite content (Fo=Mg/(Fe+Mg) of 80 appeared since the 1914–1915 eruption. Based on the bulk rock and mineral chemistry, Nakagawa *et al.* (2011) proposed that binary mixing of dacitic and andesitic endmember magmas formed the eruptive products of the 1471–1476 and 1779–1782 eruptions, while contribution from a third basaltic magma containing olivine phenocrysts is required to explain the compositional trend of erupted magmas since the 1914–1915 eruption. Silicic and mafic magmas of Yanagi *et al.* (1991) correspond to dacitic and andesitic endmember magmas of Nakagawa *et al.* (2011), respectively.

In this study, orthopyroxene phenocrysts with Mg# = ~65 and clinopyroxene phenocrysts with Mg# = ~70 have silicic melt inclusions (70–72 wt% in SiO_2_), which are supposed to correspond to the melt of the dacitic endmember magma. Clinopyroxene with Mg# = 81 includes the most mafic melt (61 wt% in SiO_2_), which is assumed to correspond to the melt composition of the andesitic endmember magma. Olivine phenocrysts with Fo_80_, which are found in the 1914–1915 lavas, scarcely contain melt inclusions large enough for EPMA analyses; thus, the melt composition of the basaltic magma is unclear. These olivine phenocrysts sometimes make an aggregate with An_90–95_ plagioclase, indicating that the basaltic magma contained An_90–95_ plagioclase and Fo_80_ olivine as phenocrysts. The Fo_80_ olivine phenocrysts have thin reaction rims of orthopyroxene and magnetite (less than 10 μm thick).

**Supplementary References**

Kobayashi, T. *et al.* *Geological Map of Sakurajima Volcano (2nd edition)*. Geological Survey of Japan (in Japanese with English abstract, 2013).

Koto, B. The great eruption of Sakura-jima in 1914. *J. Coll. Sci., Imp. Univ. Tokyo* **38,** 1–237 (1916).

Matsumoto, A., Nakagawa, M., Amma-Miyasaka, M. & Iguchi, M. Temporal variations of the petrological features of the juvenile materials during 2006 to 2010 from Showa crater, Sakurajima volcano, Kyushu, Japan. *Bull. Volcano. Soc. Japan* **58,** 191–212 (2013).

Matsumoto, K. & Nakamura, M. Syn-eruptive desulfidation of pyrrhotite in the pumice of the Sakurajima 1914–15 eruption: Implication for potential magma ascent rate meter. *J. Mineral. Petrol. Sci.* **107,** 206–211 (2012).

Miwa, T. & Geshi, N. Decompression rate of magma at fragmentation: Inference from broken crystals in pumice of vulcanian eruption. *J. Volcanol. Geotherm. Res.* **227–228,** 76–84 (2012).

Mujin, M. & Nakamura, M. A nanolite record of eruption style transition. *Geology* **42,** 661–614 (2014).

Mujin, M., Nakamura, M. & Miyake, A. Eruption style and crystal size distributions: Crystallization of groundmass nanolites in the 2011 Shinmoedake eruption. *Am. Mineral.* **102,** 2367–2380 (2017).

Nakagawa, M., Matsumoto, A., Miyasaka, M. & Iguchi, M. Change of mode of eruptive activity and the magma plumbing system of Sakurajima volcano since the 20th century. In *Study on Preparation Process of Volcanic Eruption Based on Integrated Volcano Observation 2010*, 85–94 (Sakurajima Volcano Research Center, in Japanese with English abstract, 2011).

Omori, F. The Sakura-jima eruptions and earthquakes I and II. *Bull. Imp. Earthq. Invest. Comm*. **8,** 1–34 and 35–179 (1914, 1916).

Takahashi, M. *et al.* Temporal variation for magmatic chemistry of the Sakurajima volcano and Aira caldera region, southern Kyushu, southwest Japan since 61 ka and its implications for the evolution of magma chamber system. *Bull. Volcanol. Soc. Japan* **58,** 19–42 (2013).

Yamanoi, Y., Takeuchi, S., Okumura, S., Nakashima, S. & Yokoyama, T. Color measurements of volcanic ash deposits from three different styles of summit activity at Sakurajima volcano, Japan: Conduit processes recorded in color of volcanic ash. *J. Volcanol. Geotherm. Res.* **178,** 81–93 (2008).

Yanagi, T., Ichimaru, Y. & Hirahara, S. Petrochemical evidence for coupled magma chambers beneath the Sakurajima volcano, Kyushu, Japan. *Geochem. J.* **25,** 17–30 (1991).

Yasui, M., Takahashi, M., Ishihara, K. & Miki, D. Records on the 1914-1915 eruption of Sakurajima volcano, Japan. *Proc. Inst. Natural Sci., Nihon University* **41,** 75–107 (in Japanese with English abstract, 2006).
